# Effectiveness of Therapeutic Exercise in Fibromyalgia Syndrome: A Systematic Review and Meta-Analysis of Randomized Clinical Trials

**DOI:** 10.1155/2017/2356346

**Published:** 2017-09-20

**Authors:** M. Dolores Sosa-Reina, Susana Nunez-Nagy, Tomás Gallego-Izquierdo, Daniel Pecos-Martín, Jorge Monserrat, Melchor Álvarez-Mon

**Affiliations:** ^1^Department of Medicine and Medical Specialty, Faculty of Medicine and Health Sciences, University of Alcalá, Alcalá de Henares, Spain; ^2^Immune System Diseases-Rheumatology and Oncology Service, University Hospital “Príncipe de Asturias”, Alcalá de Henares, Madrid, Spain; ^3^Department of Nursing and Physiotherapy, Faculty of Medicine and Health Sciences, University of Alcalá, Alcalá de Henares, Spain

## Abstract

**Objective:**

The aim of this study was to summarize evidence on the effectiveness of therapeutic exercise in Fibromyalgia Syndrome.

**Design:**

Studies retrieved from the Cochrane Plus, PEDro, and Pubmed databases were systematically reviewed. Randomized controlled trials and meta-analyses involving adults with fibromyalgia were included. The primary outcomes considered in this systematic review were pain, global well-being, symptoms of depression, and health-related quality of life.

**Results:**

Effects were summarized using standardized mean differences with 95% confidence intervals using a random effects model. This study provides strong evidence that physical exercise reduces pain (−1.11 [95% CI] −1.52; −0.71; overall effect *p* < 0.001), global well-being (−0.67 [95% CI] −0.89, −0.45; *p* < 0.001), and symptoms of depression (−0.40 [95% CI] −0.55, −0.24; *p* < 0.001) and that it improves both components of health-related quality of life (physical: 0.77 [95% CI] 0.47; 1.08; *p* < 0.001; mental: 0.49 [95% CI] 0.27; 0.71; *p* < 0.001).

**Conclusions:**

This study concludes that aerobic and muscle strengthening exercises are the most effective way of reducing pain and improving global well-being in people with fibromyalgia and that stretching and aerobic exercises increase health-related quality of life. In addition, combined exercise produces the biggest beneficial effect on symptoms of depression.

## 1. Introduction

Fibromyalgia Syndrome (FMS) is a rheumatic disease of unknown etiology [1] which is characterized by widespread pain and associated with multiple other symptoms including fatigue, anxiety, and depression [2]. The global mean prevalence of FMS in the general population is 2.7% with a female-to-male ratio of 3 : 1 [3] and the diagnosis is most often made in the middle age [4].

There is evidence from randomized controlled trials (RCTs) that some treatments, for example, pharmacotherapy, patient education, behavioral therapy, and physiotherapy, are effective in reducing symptoms [5]. Physiotherapy techniques used with this patient group include massage therapy, kinesiotherapy, electrotherapy, hydrotherapy, and therapeutic exercise (TE). TE seems to be effective, but there is no consensus on the type, frequency, duration, and intensity of physical activity which is beneficial to this population [6].

The aims of TE include the prevention of dysfunction and the development, restoration, or maintenance of strength, aerobic resistance, mobility, flexibility, coordination, balance, and functional abilities [7–9].

Methods used in TE include aerobic training, coordination and balance training, posture stabilization, body mechanics, flexibility exercises, gait training, relaxation techniques, and muscle strengthening exercises [10–12].

The aim of this meta-analysis was to summarize evidence on the effectiveness of therapeutic exercise in FMS.

## 2. Methods

This review was performed according to the Preferred Reporting Items for Systematic Reviews and Meta-Analysis (PRISMA) statement [13] and the recommendations of the Cochrane Collaboration [14, 15].

### 2.1. Data Sources and Searches

A systematic review of publications retrieved from the Cochrane Plus, PEDro, and Pubmed databases was performed. A manual search of the journals FisioterapiaandCuestiones de Fisioterapia was also carried out. The search strategy is detailed in Additional File (see Supplementary Material available online at https://doi.org/10.1155/2017/2356346). Only fully published material in Spanish or English was reviewed. The keywords used in database searches were “fibromyalgia”, “physical activity”, “exercise”, and “exercise therapy”. The search strategy was adapted as necessary for each database. This comprehensive search was performed from April 2016 to May 2017.

### 2.2. Study Selection

The search was conducted by two authors (DS, SN) who screened the titles and abstracts of potentially eligible studies. DS and SN also independently examined the full text of articles which passed the initial screening in order to determine whether they met the selection criteria. Cases where there was a discrepancy between the two reviewers were reevaluated and a consensus decision was achieved by discussion.

### 2.3. Eligibility Criteria

#### 2.3.1. Type of Study

RCTs comparing types of therapeutic exercise or comparing therapeutic exercise with a control group receiving another intervention or standard care were included.

#### 2.3.2. Participants

Studies with participants older than 18 years, diagnosed with FMS in the absence of significant comorbidity, were included.

#### 2.3.3. Type of Intervention

Studies using aerobic, strengthening, or stretching exercises or a combination of these were considered. Studies of exercise interventions based on activities such as yoga or tai-chi were excluded.

#### 2.3.4. Comparisons

All included studies compared the effect of at least one type of exercise with a control treatment, either another form of physical activity or standard care.

#### 2.3.5. Outcomes Measures

All included studies assessed at least one key domain of FMS symptoms (pain; symptoms of depression; global well-being; health-related quality of life (HRQOL)).

### 2.4. Data Extraction

Two authors (DS, TG) extracted the data independently using standard extraction forms. Data collected included participants, sample sizes, duration of studies, interventions, outcomes, results, and methods to measure outcomes. Discrepancies were rechecked and consensus was achieved by discussion.

Data extracted after treatment were considered an experimental group and values presented by the patients before treatment as a control group. When two different treatments were compared in the same study they were treated as independent studies for the purposes of the meta-analysis, because the aim of this study was to compare the effects of various therapies.

On the other hand, for each variable two subgroups were differentiated depending on whether the analysis by intention-to-treat or per protocol was performed in the study. When standard deviations (SDs) were not reported in the publication, they were calculated based on what was published from *t*-values, confidence intervals, or standard errors or used the mean of the SDs from other studies using the same outcome scale.

### 2.5. Data Items

The following items were extracted: author/year, design of the study, participants, interventions, comparisons, outcomes studied in this meta-analysis, and conclusions.

When researchers reported more than one indicator for an outcome a predefined order of preference for analysis was used. These preferences were predefined according to the specificity of each outcome measure (in descending order):

*Pain*. Visual Analogue Scale (VAS), VAS, from Fibromyalgia Impact Scale (FIQ), and Multidimensional Pain Inventory subscale

*Global Well-Being*. FIQ total score

*Symptoms of Depression*. Beck Depression Inventory (BDI), Hospital Anxiety and Depression Scale (HAD), and VAS from FIQ

*HRQOL*. Total SF-36 questionnaire (SF-36) score.

### 2.6. Risk of Bias within Studies and Methodological Quality

Two pairs of reviewers (DS, SN and TG, DP) worked independently to assess the methodological quality in accordance with the CONSORT 2010 [16] statement (Consolidated Standards of Reporting Trials), which contains 25 items scored as zero or one. Only studies that scored over 15 on the CONSORT checklist were included. In addition, the Cochrane Collaboration's tool was used to assess the risk of bias. Sequence generation, allocation concealment, blinding, completeness of outcome data, and absence of selective outcome reporting were also assessed. Risk of bias was classified as low, unclear, or high in each domain.

### 2.7. Data Synthesis and Analysis

#### 2.7.1. Summary Measures

The meta-analysis was conducted using the Review Manager Analysis software (RevMan 5.3) from the Cochrane Collaboration. Standardized mean differences (SMDs) were calculated from the means and SMDs for each intervention. The SMD used in RevMan software is the measure of effect size known as Hedge's (adjusted) *g*, which is the difference between the 2 means divided by the pooled SD, with a correction for small sample bias. Hedge's (adjusted) *g* was chosen because most of the studies included in this meta-analysis were small (<40 subjects per group). As it uses quantitative measures and continuous variable, the statistical analysis method used was the inverse variance [15].

The combined results were assessed using a random effects model, which is more conservative than a fixed effects model and incorporates both within- and between-study variance. Cohen's *g* was used to evaluate the magnitude of the effect size, calculated as SMD, using the following criteria: *g* > 0.2 to 0.4 small effect size; *g* > 0.4 to 0.8 medium effect size; *g* > 0.8 large effect size. Overall effects were assessed using the *Z* statistic; *p* < 0.05 was the criterion for rejection of the null hypothesis, that is, concluding that a systematic effect had been demonstrated [17]. The results of the meta-analysis were classified using the following modified level of evidence descriptors: strong = consistent results in at least two RCTs of moderate quality; moderate = consistent results in at least two low quality RCTs and/or one moderate quality RCT; limited = results in low quality RCTs; conflicting = inconsistent results in multiple RCTs; without evidence = no RCT evidence available.

#### 2.7.2. Planned Methods of Analysis

Heterogeneity was assessed using the *I*^2^ statistic: *I*^2^ < 40% heterogeneity might not be important; *I*^2^ = 30–60% may represent moderate heterogeneity; *I*^2^ = 50–90% may represent substantial heterogeneity; *I*^2^ = 75–100% may represent considerable heterogeneity. The significance of *I*^2^ depends on the magnitude and the impact of heterogeneity tests (e.g., Chi-squared test). Cochran's *Q* statistic was also calculated. This statistic is associated with the chi-squared statistic of heterogeneity with *k* − 1 degrees of freedom, where *k* is the number of included studies. If *Q* is significant, *p* < 0.10, it is likely that at least one of the included studies is different from the others.

In the random effects model tau^2^ (*t*^2^) is also used to estimate the variance in the distribution of effects across studies. If *t*^2^ = 0 the results of random effects meta-analysis would be almost identical to those of a fixed effects analysis, indicating that there is no heterogeneity [15].

#### 2.7.3. Sensitivity Analysis

In order to examine the influence of individual studies on the overall results, pooled analyses were conducted with each study individually deleted from the model. This enabled us to investigate causes of heterogeneity [15].

#### 2.7.4. Subgroup Analysis

The effects of the various types of exercise (aerobic, strengthening, stretching, and combined) were also analyzed separately.

#### 2.7.5. Risk of Bias across Studies

Potential publication bias was assessed by visually inspecting the funnel plot (plots of effect estimates against standard error) produced by the RevMan Analysis software. Publication bias tends to result in asymmetrical funnel plots [15, 18]. Data on all variables from intention-to-treat analysis were combined to produce the funnel plot.

## 3. Results

### 3.1. Study Selection

**Figure 1:**
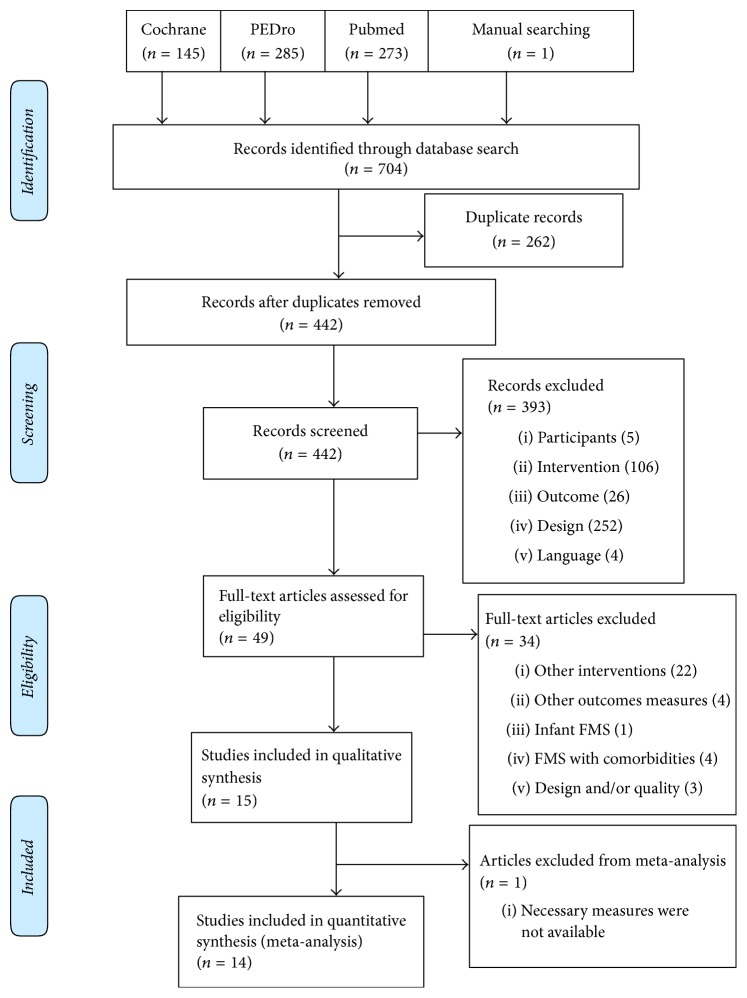
Flow diagram of procedure for selection of studies.

The literature search produced 704 citations, of which 262 were double hits (studies found in at least two data sources). Screening of title and abstracts resulted in exclusion of 393 studies. After reading the full text of the remaining articles, 33 studies were excluded. 16 RCTs were included in the qualitative synthesis, but only 14 were included in the quantitative analyses because the required measures were not available for 2 studies (Figure 1).

### 3.2. Study Characteristics

General characteristics of included studies are detailed in Table 1. One study was conducted in Norway [19], one was in United Kingdom [20], two were in Brazil [21, 22], three were in Spain [23–25], three were in the United States [26–28], three were in Sweden [29–31], and two were in Turkey [32, 33]. Patients were recruited by a fibromyalgia association in two studies [19, 23], by local newspaper advertisement in three [29–31], through a rehabilitation center in three [26, 28, 30], by a support group in two [24, 25], and through a hospital rheumatology service in four [21] and one study did not specify how participants were recruited [22]. Analysis by intention-to-treat was performed in ten studies [19–21, 24–26, 28–31].

### 3.3. Participants

The number of groups compared in the studies varied: one study compared four groups (two exercise groups, one self-help course group, and a combination of exercise and self-help course group) [28], two studies compared three groups [19, 21, 24] (two interventions groups and a control group), two studies compared one type of exercise with a control group [20, 31], and four studies [26, 27, 32, 33] compared two different types of exercise without a control group; one of them was identified as an equivalence study [26]. In total 715 participants were studied before and after treatment. Three studies included men in the sample, in total 15 men of 165 patients. Almost all the participants were women (*n* = 700, 97.90%); there were 15 (2.10%) male participants. The average age of participants was 42.36 years.

### 3.4. Interventions

Nine studies [19, 20, 22, 26, 28, 29, 32–34] investigated the effects of aerobic exercise, either walking [21, 24, 28, 30], exercise on a cycloergometer [20, 26], or exercise on a treadmill [20, 32, 33]. Seven studies [21, 22, 26, 27, 29, 31, 33] investigated muscle strengthening and two studies investigated stretching [22, 27]. Four studies [23–25, 28] investigated the effects of a combination of types of exercise (aerobic, strengthening, and stretching exercises). Control groups performed relaxation exercises [20, 29, 31], balance exercises [32], and low intensity aerobic exercise [30] or received standard care [19, 21, 23, 25]. However, in this meta-analysis data after treatment were considered an experimental group and values presented by the patients before treatment as a control group. Seven studies compared two exercise treatments (aerobic versus strengthening; [21, 33] combined versus aerobic [24, 25]; strengthening versus stretching [22, 27]; and aerobic versus strengthening [26]), as well as comparing both exercise treatments with a control condition; these studies thus had three groups [21, 24]. In the remaining seven studies, one type of exercise treatment was compared with a control group [19, 20, 23, 25, 29–31].

### 3.5. Variables

There was much variability in the outcome measures used in the included studies. Pain intensity was assessed using the VAS in five studies [19, 21, 22, 26, 33], and two used the SF-36 pain subscale [23, 25], one the Multidimensional Pain Inventory [26], and three the FIQ pain scale [27, 28, 30].

FMS severity was evaluated using the FIQ in eleven studies [20–25, 27–30, 32]. HRQOL was assessed with the SF-36 in seven studies [21–25, 29, 33]; symptom of depression was evaluated with the BDI in four studies [22, 24, 25, 28], by Hospital Anxiety and Depression Scale in three [30, 31, 33], and by VAS in one [19].

### 3.6. Risk of Bias within Studies and Methodological Quality

After critical review of each study included, it was concluded that all the studies included in this exceeded minimum thresholds for methodological and scientific quality.

**Figure 2:**
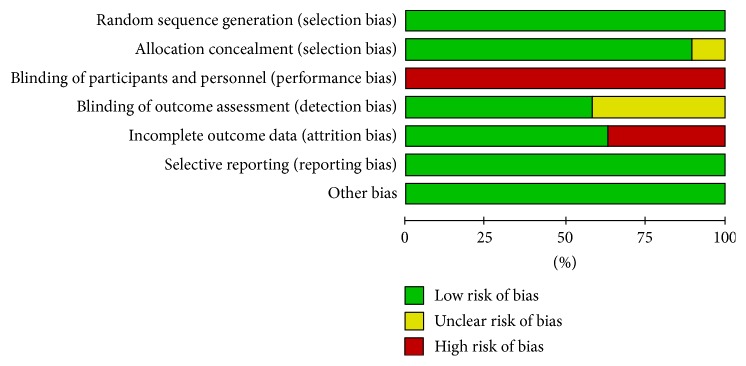
Risk of bias graph: review authors' judgements about each risk of bias item presented as percentages across all included studies.

However, since it is impossible to blind participants to group assignment in exercise intervention protocols, all studies were considered to be at a high risk of bias with respect to blinding of participants and personnel (Table 2) (Figure 2).

### 3.7. Results of Individual Studies

**Figure 3:**
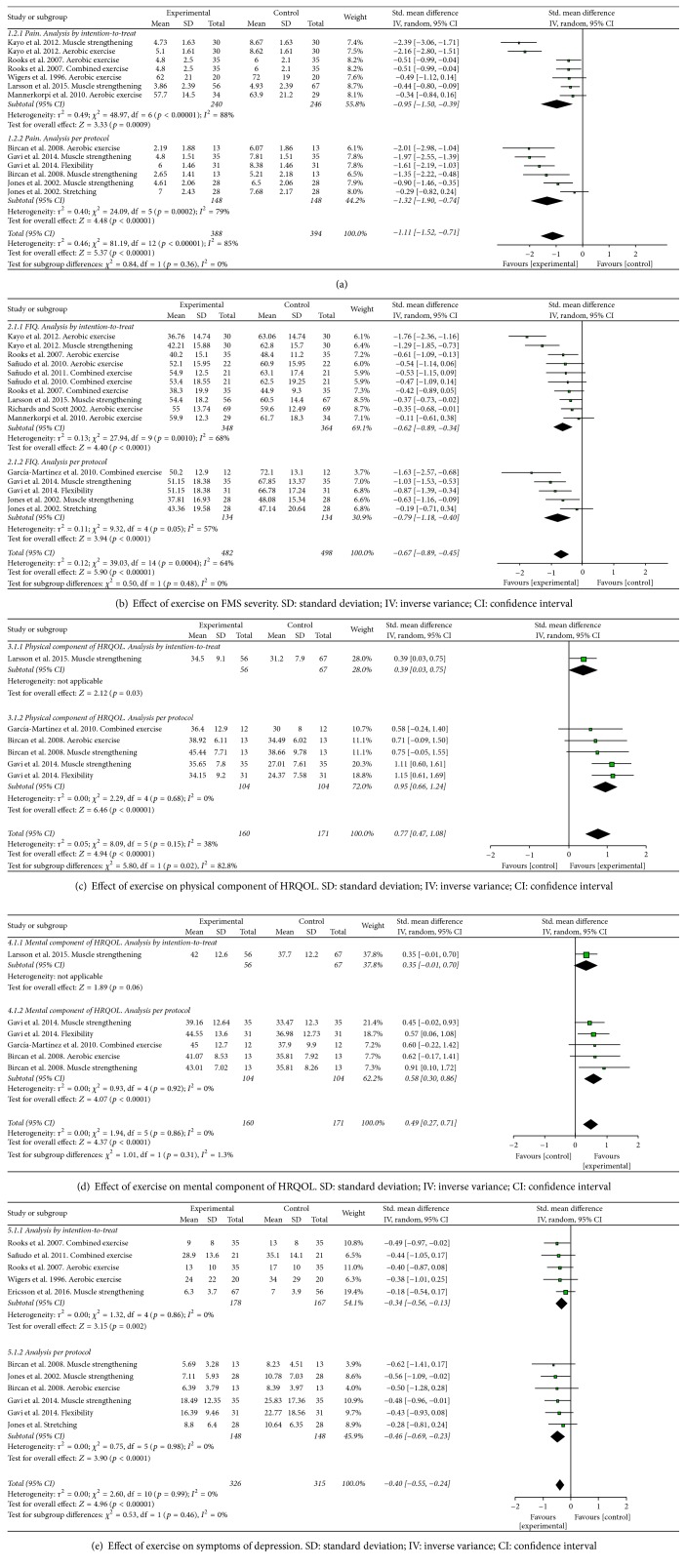


The means, SDs, sample sizes, and effect estimates for all studies can be seen in the forest plot (Figures 3(a)–3(e)).

### 3.8. Synthesis Results

Results are reported as SMDs (95% confidence interval). In the case of pain scales, FMS impact, and depression a negative result indicates that the treatment produced an improvement in patients' condition; but the opposite is true for HRQOL, where a positive effect of treatment is indicated by a positive SMD. There is strong evidence from intention-to-treat and per protocol analysis that exercise reduces pain (−1.11 [95% CI] −1.52, −0.71; overall effect *p* < 0.001), severity of FMS (−0.67 [95% CI] −0.89, −0.45; *p* < 0.001), and symptoms of depression (−0.40 [95% CI] −0.55, −0.24; *p* < 0.001) and increases both the physical and mental component of HRQOL (physical: 0.77 [95% CI] 0.47, 1.08; *p* < 0.001; mental: 0.39 [95% CI] 0.52, 0.27; *p* < 0.001). Values of Cohen's g suggested that exercise had a large effect on pain, medium effect on FMS impact and both the physical and mental component of HRQOL, and small effect on symptoms of depression.

### 3.9. Subgroup Analysis

There was strong evidence on the basis of intention-to-treat and per protocol analysis that* aerobic exercise* produces a large reduction in pain (−1.05 [95% CI] −1.78, −0.33; overall effect *p* < 0.001), small effect on symptoms of depression (−0.39 [95% CI] −0.77, −0.01; overall effect *p* < 0.05), and a medium reduction in FMS severity as assessed by FIQ (−0.65 [95% CI] −1.14, −0.16; overall effect *p* < 0.02). However, there was moderate evidence from per protocol analysis that aerobic exercise does not improve both the physical and mental component of HRQOL (0.71 [95% CI] −0.09, 1.50; overall effect *p* > 0.05 and 0.71 [95% CI] −0.14, 1.45; overall effect *p* > 0.05, resp.). There was strong evidence from intention-to-treat and per protocol analysis that* muscle strengthening* decreases pain (−1.39 [95% CI] −2.16, −0.62; overall effect *p* < 0.001), produces a reduction in FMS severity (−0.84 [95% CI] 1.23, −0.45; overall effect *p* < 0.001), and has a beneficial effect on symptoms of depression (−0.37 [95% CI] −0.61, −0.13; overall effect *p* < 0.02). In addition, muscle strengthening improves both components of HRQOL (physical: 0.72 [95% CI] 0.23, 1.21; overall effect *p* < 0.02 and mental: 0.44 [95% CI] 0.17, 0.71; overall effect *p* < 0.02). The effect size was large for the variables pain and FMS severity, medium for the physical and mental component of HRQOL, and small for the symptoms of depression. There was a strong evidence from per protocol analysis that* stretching exercises* are not effective in decreasing pain (−0.94 [95% CI] −2.24, 0.35; overall effect *p* > 0.05) and do not produce a reduction in FMS severity (0.53 [95% CI] −1.19, 0.14; overall effect *p* > 0.05). There was moderate evidence that stretching exercises improve both components of HRQOL (physical: 1.15 [95% CI] 0.61, 1.69; overall effect *p* < 0.001 and mental: 0.57 [95% CI] 0.06, 1.08; overall effect *p* < 0.05). In addition, there was strong evidence from per protocol analysis that this type of exercise reduces symptoms of depression (−0.36 [95% CI] −0.72, −0.00; overall effect *p* < 0.05). The effect size was large for the variable physical component of HRQOL, medium for mental component of HRQOL, and small for symptoms of depression.

There was moderate evidence on the basis of intention-to-treat analysis that* combined exercise* produces a large decrease in pain (−0.51 [95% CI] −0.99, −0.44; overall effect *p* < 0.05). In addition, there was strong evidence that this type of exercise produces a medium reduction in symptoms of depression (−0.47 [95% CI] −0.85, −0.10; overall effect *p* < 0.05). There was strong evidence from intention-to-treat and per protocol analysis that combined exercise produces a medium reduction in FMS severity (−0.64 [95% CI] −1.06, −0.22; *p* < 0.02) The effect on HRQOL was only assessed by per protocol analysis; this provided that this type of exercise does not improve HRQOL (physical: 0.58 [95% CI] −0.24, 1.40; overall effect *p* > 0.05 and mental 0.60 [95% CI] −0.12, 1.42; overall effect *p* < 0.05, physical HRQOL (−0.58 [95% CI] −0.24, 1.40; *p* > 0.05) and mental HRQOL (0.60 [95% CI] −0.22, 1.42; *p* > 0.05)). The effect sizes were large for pain and medium for symptoms of depression, FMS severity, and physical and mental HRQOL.

### 3.10. Sensitivity Analysis

Heterogeneity (measured as *I*^2^) in data on pain from both intention-to-treat and per protocol analysis was eliminated by excluding from analysis the studies by Kayo et al. and Jones et al. [21, 27]. Once these studies had been eliminated, there was still strong evidence that exercise produces a large reduction in pain (−0.97 [95% CI] −1.39, −0.55; overall effect *p* < 0.001). Using the same study-by-study exclusion procedure it was found that heterogeneity in data on FMS severity was eliminated by excluding the same studies. Eliminating the Kayo et al. and Jones et al.'s studies [21, 27] the effect size was increased and there was still strong evidence that exercise produces a large decrease in FMS severity (−1.04 [95% CI] −1.37, −0.70; overall effect *p* < 0.001).

The remaining data on outcome variables were homogeneous and therefore other sensitive analyses were not necessary.

### 3.11. Risk of Bias across Studies

**Figure 4:**
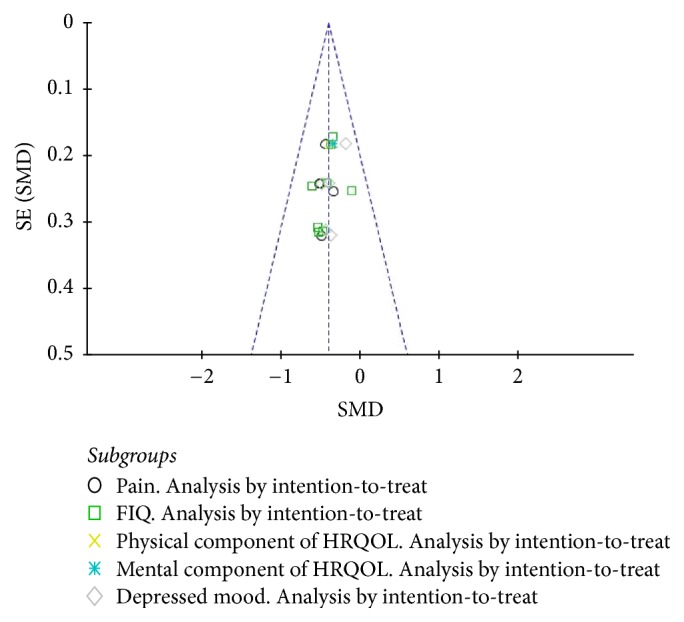
Funnel plot of publication bias. SE: error standard; SMD: standardized mean difference; FIQ: fibromyalgia impact questionnaire; HRQOL: health-related quality of life.

On visual inspection, the funnel plot of posttreatment outcomes was symmetrical and there was thus no evidence of publication bias. Due to heterogeneity produced by Kayo et al.'s study, data from this study were excluded for this analysis (Figure 4).

## 4. Discussion

The use of various exercise interventions in the studies presented above notwithstanding any physical activity is damaging for people with FMS.

This is the first meta-analysis to assess the most effective exercise for improving some symptoms or conditions in fibromyalgia.

Aerobic exercise for 30 to 60 minutes at an intensity of 50–80% of maximum heart rate 2 or 3 times per week for a period of 4–6 months and muscle strengthening exercises (1 to 3 sets of 8–11 exercises, 8–10 repetitions with a load of 3.1 kg or 45% of 1 repetition maximum (RM)) seem to be most effective in decreasing the pain and severity of FMS. Stretching the major muscle groups and aerobic exercise can improve the physical and mental component of HRQOL, respectively. Combined exercise programs consisting of aerobic exercise, muscle strengthening, and stretching exercises performed for 45–60 minutes 2 or 3 times per week for 3–6 months seem to be the most effective in reducing the symptoms of depression. The findings of this research are consistent with two previous equivalence studies [21, 26] which concluded that aerobic and strengthening exercise have similar effects on pain intensity and FMS severity. In addition, like in this study, Kayo et al. [21] and Bircan et al. [33] also found that both aerobic and strengthening exercise were equally effective in improving HRQOL. However, this meta-analysis found that stretching exercise produces a greater improvement in the physical component of HRQOL than the rest of types of exercise that were studied whereas aerobic and combined exercise seem to be better at improving mental quality of life. Kayo et al. [21] noted that after 12 weeks without exercise the group who had performed muscle strengthening exercises had experienced recurrence of symptoms, whereas the beneficial effects of aerobic exercise persisted longer.

The results of other meta-analyses were also considered. In 2010, Häuser et al. [34] compared various types of aerobic exercise and found that exercising in the water and on dry land was similarly effective, this one being mild to moderate intensity, with a frequency of 2-3 times per week for 20–30 minutes at least for 4 weeks. Like García-Martínez et al. [23] Häuser et al. concluded that for the effects on physical condition and depression to persist the patients must maintain the exercise regime and therefore need to be motivated [34].

In another meta-analysis published by Kelley et al. [35] in the same year, seven studies were collected, to investigate the effects of physical activity, consisting of 15–60-minute sessions of aerobic and/or muscle strengthening exercises 2 or 3 times per week for a period of 12–23 weeks. The meta-analysis by Kelley et al. [35], like this study, suggested that physical activity improves the general well-being of women with FMS.

This meta-analysis had several limitations, one of which is the sample size of the included studies; most used relatively few participants. In addition, studies included in this meta-analysis were performed predominantly in women due to the fact that fibromyalgia is a syndrome with a significant female predominance [3]. This could be a limitation of the present study because it is not known whether the results obtained could be extrapolated to the male population suffering from fibromyalgia.

Unlike pharmacological studies, which are easily blinded, behavioral and physical treatment requiring the active participation of patients is virtually impossible to be blinded.

It is also important to take into account the heterogeneity of the studies, primarily due to the inclusion of the studies of Kayo et al. and Jones et al. [21, 27]. This heterogeneity should be taken into consideration when drawing conclusions from the analysis of this study.

Another important limitation is that each type of therapeutic exercise was investigated in only a small number of studies. Aerobic exercise is the most commonly studied type of exercise treatment for FMS.

## 5. Conclusions

Exercise is beneficial for people with FMS but it is unable to draw any conclusions about what type of exercise is most effective because not enough studies were included in this meta-analysis. There is some evidence to suggest that muscle strengthening and aerobic exercise are most effective in reducing the pain and severity of the disease whilst stretching and aerobic exercise produce the biggest improvements in HRQOL. Combined exercise is the most effective way of reducing symptoms of depression. Although there is still no consensus, it seems that 2 or 3 sessions of mild to moderate intensity physical activity lasting 30–45 minutes each are effective.

It would be interesting to conduct primary research into the type of exercise likely to yield the highest rate of adherence to an exercise treatment regime using a larger sample, because for the effects to be sustained the patients must continue with regular physical activity.

It would also be interesting to investigate whether group and individual physical activity have similar psychological benefits.

## Supplementary Material

Search strategy.

## Figures and Tables

**Table 1:** General characteristics of included studies.

Author/year	Design	Participants	Intervention/comparison	Outcomes	Conclusions
Wigers et al. 1996 [19]	RCT	AE: 18 women and 2 men (*n* = 20) with FMS SMT: 18 women and 2 men (*n* = 20) with FMS CG: 19 women and 1 man (*n* = 20) with FMS	*Intervention:* aerobic exercise (AE) and stress management treatment (SMT) *Comparison:* usual care (CG) *Duration:* AE: 3 days a week (45 minutes) for 14 weeks. SMT*:* 2 days a week (90 minutes) for 6 weeks and 1 day a week (90 minutes) for 8 weeks	(i) Pain (ii) Symptoms of depression	Aerobic exercise was the overall most effective treatment.

Jones et al. 2001 [27]	RCT	EG: 28 women with FMS CG: 28 women with FMS	*Intervention:* muscle strengthening (EG) *Comparison:* stretching exercises (CG) *Duration:* 2 days a week (60 minutes) for 12 weeks	(i) Pain (ii) FMS impact	Muscle strengthening produces an improvement in overall disease activity.

Richards and Scott 2002 [20]	RCT	EG: 62 women and 5 men (*n* = 67) with FMS CG: 64 women and 5 men (*n* = 69) with FMS	*Intervention:* aerobic exercise (EG) *Comparison:* relaxation (CG) *Duration:* EG: 2 days a week (12–50 minutes) for 12 weeks. CG: 2 days a week (60 minute) for 12 weeks	(i) FMS impact	Aerobic exercise is an effective treatment for FMS.

Rooks et al. 2007 [28]	RCT	AE: 35 women with FMS ST: 35 women with FMS FSHC: 27 women with FMS ST-FSHC: 38 women with FMS	*Interventions:* aerobic exercise (AE), strength training, aerobic exercise and stretching (ST), fibromyalgia self-help course (FSHC), and a combination of ST and FSHC (ST-FSHC) *Duration:* AE and ST: 2 days a week (60 minutes) for 16 weeks. FSHC: 120 minutes every two weeks	(i) Pain (ii) FMS impact	Progressive walking, simple strength training movements, and stretching activities improve functional status, key symptoms, and self-efficacy in women with FMS.

Bircan et al. 2008 [33]	RCT	AE: 13 women with FMS SE: 13 women with FMS	*Interventions:* aerobic exercise (AE) and strengthening exercise (SE) *Duration:* 3 days a week (30–40 minutes) for 8 weeks	(i) Pain (ii) Symptoms of depression	AE and SE are similarly effective at improving symptoms, depression, and quality of life in FMS.

García-Martínez et al. 2010 [23]	RCT	EG: 14 women with FMS CG: 14 women with FMS	*Intervention:* exercise combined protocol (aerobic, strengthening, and stretching exercises) (EG) *Comparison:* normal daily activities (CG) *Duration:* 3 days a week (60 minutes) for 12 weeks	(i) Pain (ii) HRQOL (iii) FMs impact	The GE improved quality of life, psychological state, and physical functioning.

Sañudo et al. 2010 [24]	RCT	EG1: 22 women with FMS EG2: 21 women with FMS CG: 20 women with FMS	*Interventions:* aerobic exercise and combined exercise (EG1 and EG2) *Comparison:* Normal daily activities (CG) *Duration:* GE1: 2 days a week (45–60 minutes) GE2: 2 days a week (35–45 minutes), 24 weeks	(i) FMS impact (ii) HRQOL (iii) Symptoms of depression (iv) Pain	An improvement from baseline in total FIQ score was observed in the exercise groups and was accompanied by decreases in BDI scores. Relative to nonexercising controls, CE evoked improvements in the SF-36 physical functioning and bodily pain domains and was more effective than AE for evoking improvements in the vitality and mental health.

Mannerkorpi et al. 2010 [30]	RCT	EG: 34 women with FMS CG: 33 women with FMS	*Intervention:* Nordic walking moderate to high intensity (EG) *Comparison:* low intensity walking (CG) *Duration:* EG: 2 days a week (10–20 minutes), 15 weeks. CG: 1 day a week	(i) Pain (ii) FMS impact	The Nordic walking group had better FIQ physical scores.

Sañudo et al. 2011 [25]	RCT	EG: 18 women with FMS CG: 20 women with FMS	*Intervention:* combined exercise (aerobic, strength, and flexibility) (EG) *Comparison:* routine care (CG) *Duration:* 2 days a week (45–60 minutes) for 24 weeks	(i) HRQOL (ii) FMS impact (iii) Symptoms of depression	A combined program of long-term exercise improves psychological and health status by increasing the quality of life.

Kayo et al. 2012 [21]	RCT	WPG: 30 women with FMS SMG: 30 women with FMS GC: 30 women with FMS	*Interventions:* walking program (WPG) and muscle strengthening (SMG) exercises *Comparison:* medication only or conventional treatment (CG) *Duration:* WPG and SMG: 3 days a week (60 minutes) for 16 weeks	(i) Pain (ii) FMS impact (iii) HRQOL (iv) Use of drugs	Both modalities (WP and PSM) provided better pain relief for people with FMS than medication only or conventional treatment.

Hooten et al. 2012 [26]	RET	AE: 32 women and 4 men (*n* = 36) with FMS SE: 33 women and 3 men (*n* = 36) with FMS	*Intervention:* aerobic exercise (AE) and strengthening exercise (SE) *Duration:* AE: 10–30 minutes each day for 3 weeks SE: 25–30 minutes each day for 3 weeks	(i) Pain severity	Strengthening exercises and aerobic exercise are similarly effective in reducing pain intensity.

Gavi et al. 2014 [22]	RCT	MSE: 35 women with FMS SE: 31 women with FMS	*Intervention:* muscle strengthening exercise (MSE) and stretching exercises (SE) *Duration:* 2 days a week (45 minutes) for 16 weeks	(i) Pain (ii) HRQOL (iii) FMS impact (iv) Symptoms of depression	Both groups experienced a reduction in pain, which was more noticeable and had an earlier onset in the strengthening exercise group. Both groups experienced improvements in functionality, depression, and quality of life.

Duruturk et al. 2015 [32]	RCT	BE: 12 women with FMS AE: 14 women with FMS	*Intervention:* balance exercise (BE) and aerobic exercise (AE) *Duration:* 3 days a week (20–45 minutes) for 6 weeks	(i) Pain (ii) FMS impact	Both groups showed an improvement in pain intensity and FIQ functionality; there was no group difference on either measure.

Larsson et al. 2015 [29]	RCT	RE: 67 women with FMS CG: 63 women with FMS	*Intervention:* resistance exercise (RE) *Comparison:* relaxation exercises (CG) *Duration:* RE: 2 days a week (60 minutes) for 15 weeks. CG: 2 days a week (25 minute) for 15 weeks	(i) HRQOL (ii) Pain intensity (iii) FMS impact	Resistance exercise group reduced pain intensity.

Ericsson et al. 2016 [31]	RCT	EG: 67 women with FMS CG: 63 women with FMS	*Intervention:* resistance exercise (EG) *Comparison:* relaxation therapy (CG) *Duration:* 2 days a week (60 minutes) for 15 weeks	(i) Pain (ii) Symptoms depression	Resistance exercise improves some symptoms in women with FMS.

RCT: randomized clinical trial; RET: randomized equivalence trial; EG: exercise group; CG: control group; FMS: Fibromyalgia Syndrome.

**Table 2:** Risk of bias within studies.

	Wigers et al. 1996	Jones et al. 2002	Richards and Scott 2002	Rooks et al. 2007	Bircan et al. 2008	Duruturk et al. 2015	García-Martínez et al. 2012	Gavi et al. 2014	Hooten et al. 2012	Kayo et al. 2012	Larsson et al. 2015	Mannerkorpi et al. 2010	Sañudo et al. 2010	Sañudo et al. 2011	Ericson et al. 2016
Random sequence generation	Low risk	Low risk	Low risk	Low risk	Low risk	Low risk	Low risk	Low risk	Low risk	Low risk	Low risk	Low risk	Low risk	Low risk	Low risk
Allocation concealment	Low risk	Low risk	Low risk	Low risk	Risk unclear	Low risk	Low risk	Low risk	Low risk	Low risk	Low risk	Low risk	Low risk	Low risk	Low risk
Blinding of participants and personnel	High risk	High risk	High risk	High risk	High risk	High risk	High risk	High risk	High risk	High risk	High risk	High risk	High risk	High risk	High risk
Blinding of outcome assessment	Risk unclear	Low risk	Low risk	Risk unclear	Risk unclear	Low risk	Risk unclear	Low risk	High risk	Risk unclear	Low risk	Low risk	Low risk	Low risk	Low risk
Incomplete outcome data	Low risk	High risk	Low risk	Low risk	High risk	Low risk	High risk	High risk	Low risk	Low risk	Low risk	Low risk	Low risk	Low risk	Low risk
Selective reporting	Low risk	Low risk	Low risk	Low risk	Low risk	Low risk	Low risk	Low risk	Low risk	Low risk	Low risk	Low risk	Low risk	Low risk	Low risk
Other bias	Low risk	Low risk	Low risk	Low risk	Low risk	Low risk	Low risk	Low risk	Low risk	Low risk	Low risk	Low risk	Low risk	Low risk	Low risk
