# Supplementary material for: Effectiveness of Therapeutic Exercise in Fibromyalgia Syndrome: A Systematic Review and Meta-Analysis of Randomized Clinical Trials
Source: Biomed Res Int. 2017 Sep 20;2017:2356346. doi: 10.1155/2017/2356346 (PMC5632473; doi:10.1155/2017/2356346)
Supplement: Supplementary file 1 — Search strategy. [file 2356346.f1.pdf]

APPENDIX

Additional file. Search strategy.

Cochrane Plus:

- Advanced search: '((fibromialgia) AND (actividad física)):TA': 4 publications retrieved.
- Advanced search: '((fibromialgia) AND (ejercicio)):TA': 6 publications were retrieved.
- Advanced search: '((fibromyalgia) AND (exercise)):TI': 125 publications were retrieved.
- Advanced search: '((fibromyalgia) AND (exercise therapy)):TI': 10 publications were retrieved.

More searches were performed without retrieving any publications. In total we retrieved 145 publications.

In **PEDro** database: taking advantage of the simplicity with which the database can be searched we performed in English a combination of the following terms: 'fibromyalgia' to 'exercise', 'exercise therapy' or 'motor activity'; 285 publications were retrieved.

In **Pubmed**:

- Advanced search: '(fibromyalgia[MeSH Major Topic]) AND exercise[MeSH Major Topic] AND physical therapy[MeSH Major Topic]': 50 publications were retrieved.
- Advanced search: '(fibromyalgia[Title]) AND exercise[Title]': 212 publications were retrieved.
- Advanced search: '(fibromyalgia[Title]) AND exercise therapy[Title]': 6 publications were retrieved.
- Advanced search: '(fibromyalgia[Title]) AND exercise[Title]) AND physical therapy[Title/Abstract]': 5 publications were retrieved.

In total 273 publications were retrieved from this database.

A manual search of **'Cuestiones de Fisioterapia'** and **'Fisioterapia'** on the issue of the last five years using the keywords given above yielded 1 publication.
